# The incremental cost of implementing the world health organization Package of essential non-communicable (PEN) diseases interventions in Iran

**DOI:** 10.1371/journal.pgph.0000449

**Published:** 2023-02-08

**Authors:** Mehrdad Azmin, Farnam Mohebi, Moein Yoosefi, Naser Ahmadi, Saeed Shirazi, Mitra Modirian, Farshad Farzadfar

**Affiliations:** 1 Non-Communicable Diseases Research Center, Endocrinology and Metabolism Population Sciences Institute, Tehran University of Medical Sciences, Tehran, Iran; 2 Haas School of Business, University of California, Berkeley, California, United States of America; 3 Endocrinology and Metabolism Research Center, Endocrinology and Metabolism Clinical Sciences Institute, Tehran University of Medical Sciences, Tehran, Iran; Bangladesh Institute of Development Studies, BANGLADESH

## Abstract

World-Health-Organization’s PEN package proposes a minimum set of cost-effective interventions for early diagnosis and management of Non-Communicable-Disease (NCD). IraPEN (the PEN package implemented in Iran), adopted from PEN and Iran National Action Plans for NCDs, addresses challenges regarding NCD prevention and control. IraPEN was piloted in four districts of Iran. In this research, we estimate incremental per-capita cost of IraPEN program implementation in two of the pilot districts. We utilized a bottom-up, ingredient-based costing approach. Institutional expenditure data was collected via information forms. Information pertaining to personnel costs was gathered by performing task time measurements using Direct Observation Method. An individual-level survey was conducted in under-study districts to determine program coverage and its users’ demographic information via systematic random cluster sampling. Sampling of districts was based on systematic random cluster sampling. In each district, 250 families in 25 clusters proportional to urban or rural populations were randomly selected by postal codes. All family members eligible for the program were interviewed. Interviews were organized and conducted in each district by NCD experts in provincial Universities of Medical Sciences. Costs were re-categorized into fixed and variable costs based on their dependency on the program’s coverage. Fixed and variable costs were, respectively, divided by total eligible populations and covered populations in each district to calculate cost per-capita for each protocol. Total per-capita cost per-service was then calculated for each protocol and whole program by adding these figures. All costs are reported in US$ 2015–2016. The incremental costs of IraPEN implementation per user, with and without introduction cost, were US$24.90 and US$25.32, respectively. Total incremental cost per-capita for each protocol ranged between US$1.05 to US$7.45. The human resources and supplies had the highest contribution in total program cost (74.97% and 15.76%, respectively). The present study shows that IraPEN program implementation to be a high-cost package within Iranian context, that necessitates cautions in other similar contexts for implementation. It is, however, difficult to make decisions on implementation of NCD prevention and control programs purely based on their cost. Informed decision making requires assessment of a programs’ effectiveness and justifications and alterations to the current package could reduce the costs, leading to increased efficiency of the program.

## Introduction

The 2030 Agenda for Sustainable Development Goals is to reduce premature mortality from Non-Communicable Diseases (NCDs) by one third [1]. World Health Organization’s (WHO) “Package of Essential Non-Communicable Diseases Intervention for Primary Healthcare in Low-Resource Settings” (PEN) proposes a minimum set of cost-effective interventions for early diagnosis and management of NCDs [2] and is introduced as a tool to reduce the NCD-attributable premature mortality. Although the feasibility and cost-effectiveness of PEN has been assessed in a number of low-resource countries, the capacity to implement the package, its cost of implementation and effectiveness are, however, still under question. [3–16]. In other words, there is not adequate information on how the cost of implementation differs from the predictions of WHO and in what extend. The detailed information on the costs of implementing PEN package is required for the countries that are willing to adopt this package because countries need accurate estimations of cost of implementation to assess whether they should adopt the package in terms of costs and efficiency and in consideration of their resources, what are the detailed costs of implementation to configurate the package according to their resources and needs, and how to insure the sustained implementation of the practice if they decide to adopt it. This study helps feel the existing gap on the aforementioned notions by studying the case of pilot implementation of PEN in Iran and estimating the detailed cost of PEN implementation in Iran.

Due to high burden of NCDs in Iran [17], with the proportion of deaths attributable to NCDs being 82% in 2017 in Iran, Ministry of Health and Medical Education (MoHME) has developed a “National Action Plan for Prevention and Control of Non-Communicable Diseases and the related risk factors in Iran, 2015–2025” [18]. IraPEN (the PEN package implemented in Iran) is the name of the plan adopted from both WHO PEN package and National Action Plans for NCDs [2, 18]. Considering the high burden of NCDs and the financial limitations in addressing the burden of these diseases and prioritization concerns, Iranian healthcare policy makers are motivated to apply suggestions of expert organizations on efficient and effective policies and interventions. Thus, IraPEN is an attempt by healthcare policymakers and deputies to address challenges regarding NCD prevention and control in Iran by implementing a screening and referral system for the most common NCDs or with highest burden by healthcare providers at primary health care centers [19]. In general, healthcare policymakers considered IraPEN to be a comprehensive and effective program and exceeding the previously established type of practice in being comprehensive and integrated. Nevertheless, and like any other comprehensive policy, there are cost and structural concerns for upscaling the program.

IraPEN includes 5 protocols for: 1) prevention of myocardial infarction and stroke through integrated care of diabetes, hypertension, and dyslipidemia (MI & Stroke prevention); 2,3) screening of breast, cervical (female cancer prevention), and colorectal cancers (CRC prevention); 4) screening of asthma and other respiratory disorders including chronic obstructive pulmonary disease (Respiratory diseases screening); 5) Survey of NCD risk factors related to nutrition, alcohol/tobacco use, and low physical activity, followed by health education and counseling on health behaviors (NCD risk factors survey) [19]. This program was funded by MoHME and universities of medical sciences responsible for delivering healthcare programs at the district level.

There was a variety of managerial, political, and organizational challenges for the full-scale implementation of IraPEN [20]. Additionally, the upscaling and continued implementation the program required the long-term provision of financial resource. Therefore, MoHME started with piloting IraPEN in 4 districts to assess the feasibility and incremental cost of the program. In this research, we seek address the knowledge gap regarding the cost and resource use of IraPEN in the two pilot districts of Naqadeh and Shahreza from February 2016 to March 2017. The provided evidence can help with its future planning and may also be useful to other developing countries planning to implement cost-effective interventions to control the rapid emergence of NCDs and their associated complications.

## Materials and methods

IraPEN program pilot implementation was performed in four districts of Iran. The districts were chosen based on the assessment of the experts in MoHME on the following matters: 1) the set of locations being representative of all parts of the country in terms of the healthcare provision and population characteristics, 2) the respective Universities of Medical Sciences being representative of the types of practices that are performed in the medical universities of the country because medical universities are responsible for the provision of the type of the care that is prescribed by PEN package, and 3) the estimated cost of implementation based on experts’ previous experiences and opinions, and not mathematical estimations, do not exceed the budget of the pilot program. This resulted in choosing 4 districts in 4 provinces of the country.

**Fig 1:**
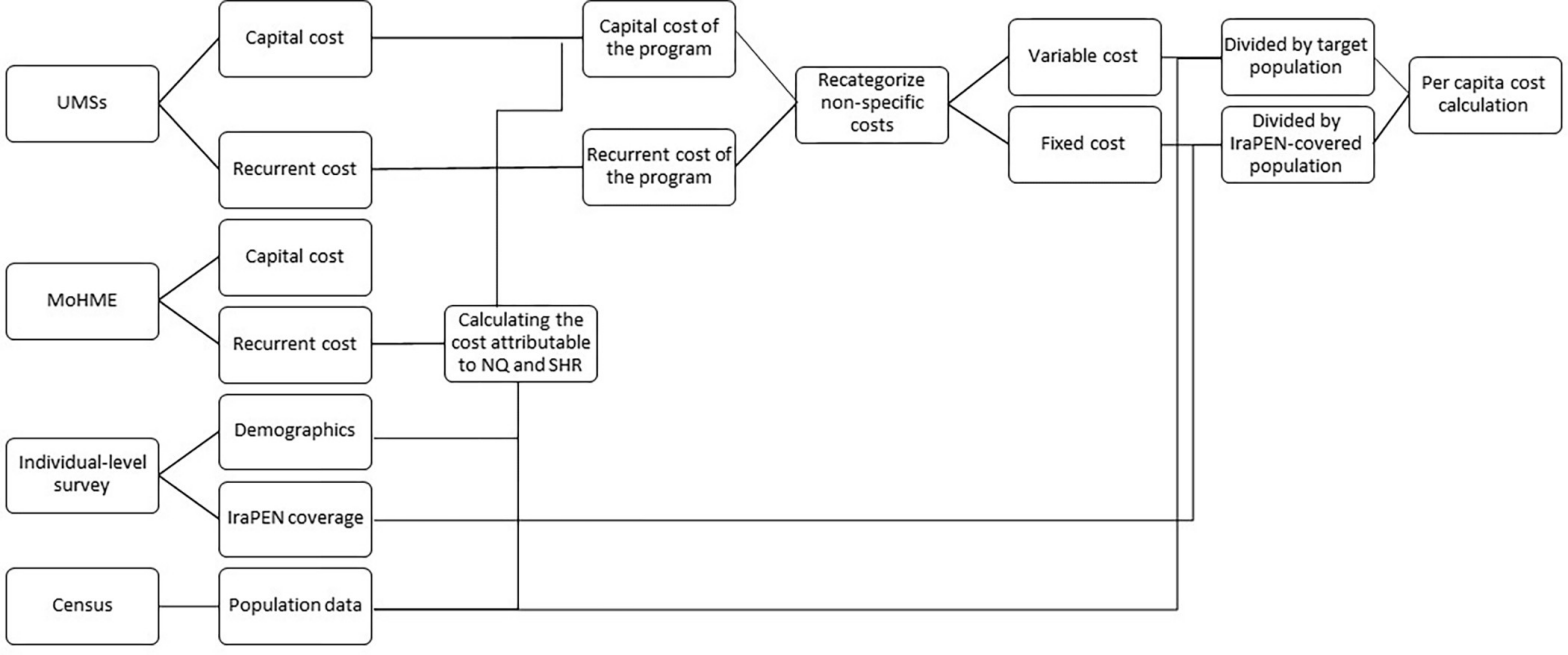
An overall view of analysis framework to calculate incremental cost of implementing the world health organization Package of essential non-communicable (PEN) diseases interventions in Iran.

In this study, we estimated the incremental per capita cost of IraPEN program pilot-implementation in Shahreza and Naqadeh districts of Iran using a bottom-up approach [21]. An overall view of analysis framework has been illustrated in Fig 1. Capital and recurrent expenditure data was initially gathered via information forms. Expenditures were then re-categorized into fixed and variable costs. Cost per capita calculated by dividing fixed and variable costs by total eligible populations and the population covered by the pilot programs respectively. Total per capita cost per service was then calculated for each protocol, by including specific and non-specific costs of the protocol, and the whole program by adding these figures. The protocols consisted of a combination of the interventions designed for: 1) prevention of myocardial infarction and stroke through integrated care of diabetes, hypertension, and dyslipidemia (MI & Stroke prevention); 2,3) screening of breast, cervical (female cancer prevention), and colorectal cancers (CRC prevention); 4) screening of asthma and other respiratory disorders including chronic obstructive pulmonary disease (Respiratory diseases screening); 5) Survey of NCD risk factors related to nutrition, alcohol/tobacco use, and low physical activity, followed by health education and counseling on health behaviors (NCD risk factors survey) [19].

### Definition of terms

- Bottom-up cost estimation: The bottom-up cost estimation method measures the detailed costs of every each and every expense, including labor, equipment, indirect, direct. In this method, the researcher estimates the expenditures at the lowest possible level of detail and then aggregate them in order to arrive at summary totals.
- Incremental cost—All expenditure introduced to health care centers or utilized specifically for implementation of IraPEN and not used for other activities of the facility. These costs comprised capital and recurrent costs.
- Capital costs–Also known as non-recurrent costs [22], defined as expenditures on on-off items. Details of cost included in this category are presented in S1 Table.
- Recurrent costs—Expenditure on consumables and to-be-regularly purchased items [22]. Details of included recurrent costs are presented in S1 Table.
- Fixed costs -Fixed costs are the expenditures which do not change with the quantity of the resources. Therefore, in this study the fixed costs did not change if the program’s coverage changed.
- Variable costs–Variable costs are in opposite side of the fixed costs in the sense that they are directly related to the scale of the program’s coverage or in other words the quantity of the goods and services produced.
- Coverage was defined as the proportion of individuals who have used at least one of IraPEN services divided by their target populations.
- Specific costs–Expenditure items attributable to a given protocol including supplies and equipment. Expenditures in this category were directly included to calculate the cost of the relevant protocol.
- Non-specific costs–Expenditures not directly related to a particular protocol. Items in this category were divided equally across protocols.

### Data sources

#### A. Institutional data collection

Institutional costs comprised expenditure by MoHME, as well as the Universities of Medical Sciences (UMSs) and District Health Networks (DHNs) responsible for delivering IraPEN services. Institutional expenditure data was collected by submitting request for information (RFI) forms to Health and Treatment deputies at MoHME and UMSs of the districts under study. The relevant authorities were asked to provide the amount spent (in Iran Rials) on each item from February 2016 to March 2017, and provide official financial receipt numbers and reports where available. Expenditure on equipment and supplies was detailed by name of items, total units purchased, unit costs, and number of units used during the period under study. The validity of the reports was assessed and approved by Health and Treatment deputies at MoHME and UMSs and by the authors. In case of any ambiguity or suspect, the authors were provided the chance to contact the responsible person for data collection. Nevertheless, the authors and the deputies did not observe and report any case of potential invalid data entry.

Information pertaining to service providers’ costs attributable to delivering IraPEN services was gathered by performing task time measurements using the Direct Observation Method (DOM). We recorded the time spent by physicians, midwives, and community health workers (CHWs) working in IraPEN delivery sites in rural and urban areas. The gathered data included details on the attending patient, appointment date, service(s) provided, and per service. This data was collected from 2 urban and 2 rural sites in each district for 10–20 patients visited at each site. The time of observing was not announced to the provision centers in advance and the observer recorded data until he/she reached at least 10 patients, continued with the cap of 20 patients. To elaborate, if the observer started observing the site in a time that 15 patients visited till the end of the working hours of the center, it was considered adequate data gathering. The observers were suggested to attend the sites when they expect to see at least 10 patients. If they failed in their attempt to gather enough data, they needed to visit the center again to continue the data gathering. Average daily time spent on managing the program on UMS, urban, and rural levels was also collected. Data on number of service providers (191 physicians, midwives, and CHWs) assigned to the delivery of IraPEN and their salaries in urban and rural areas in each district was provided by their respective UMSs and DHNs.

#### B. Individual-level data collection

An individual-level survey was conducted in the districts under study to determine program coverage and its users’ demographic information. We should not that the randomized individual level survey was only conducted in the two districts to determine coverage, effect on wider healthcare utilization, and out of pocket expenditure attributable to the program. The districts for the survey were a random choice between two options for each location. The districts of Shahreza (province of Isfahan) and Baft (province of Kerman) are very close to another both geographically and also in terms of the population characteristics, healthcare access and healthcare system attributes, and assessment of experts on the acceptability and process of IraPEN implementation and Shahreza was chosen between the two districts. This is also true for Maraqeh (province of East Azerbaijan) and Naqadeh (province of West Azarbaijan) districts and Naqadeh was chosen.

Sampling was based on systematic random cluster sampling. In each district, 250 families in 25 clusters proportional to urban or rural populations were randomly selected by postal codes. The cluster construction was as follows: first, all the recorded postal codes were two divided into two stratified clusters (urban and rural). For rural and urban areas, the required number of samples were determined proportional to the number of populations in each. Then, we needed to distribute this sample in the areas. So, we assigned numbers to the geographical blocks (as clusters) of each stratum and then randomly selected required number of blocks. Withing each block, 10 individual postal codes was chosen as each being a representation of one family. We started contacting the eligible families residing in each block and kept inviting until we reached 10 invited families in each block. All family members of a postal code eligible for IraPEN program were interviewed. Interviews were organized and conducted in each district by NCD experts in UMSs of West Azerbaijan and Isfahan. Data collection was initially carried out using paper forms and later entered into the study database by collaborators using a web portal specifically developed for this study.

### Cost estimation approach

In general, after calculation the Capital and Recurrent costs via of each protocol, costs were re-categorized into fixed and variable costs. To calculate the cost per capita, we divided fixed and variable costs were, respectively, by the total eligible populations and covered populations in each district and added up these figures to calculate the total cost of each protocol and the whole program. Therefore, we did not perform any inferential analysis for population-based estimations and we directly used and reported the collected data for cost calculations.

### A. Capital costs

- *Initial meetings*, *workshops*, *consultancies*, *supervision*, *and customization*: Expenditure on meetings, workshops, and consultancy were detailed by number of days, number of attendees, and their transportation and accommodation costs. Other hospitality costs, such as food and drinks served, were not included in these categories. Each cost item was then divided by 62, the number of UMSs in Iran, to be equally attributed to all medical universities. This step effectively only applies to expenditures by MoHME. All spending by UMSs and DHNs were categorized as “district-level”. Items in this category were also non-specific to a particular protocol and were thus equally distributed among them.
- *Equipment*: The annual cost of each equipment was calculated as their current unit cost, divided by annualization factor based on useful life time and discount rate of 10% according to capital recovery factor formula [22]. Costs of equipment were then multiplied by the number of units purchased for the program.

### B. Recurrent costs

- *Supplies*: For each district the cost of supplies was calculated for each month by dividing the total cost of supplies divided by the duration of pilot phase in months, and subsequently multiplied by 12 to calculate the annual cost of supplies.
- *Personnel (community health workers*, *midwifes*, *and physicians)*: Cost of human resources was calculated using task-times. Task-times gathered through DOM were averaged for each role to estimate time spent for each visit by provider. An estimate of daily visits per service provider was collected. These were used to calculate the average portion of working time each service provider spends on delivering IraPEN services. These results, combined with average monthly salary data obtained per service provider through Isfahan and West Azerbaijan UMSs were used to estimate the salary payments attributable to delivering IraPEN per role. The number of staffs assigned to the delivery of IraPEN were provided by UMSs and DHNs. Total expenditure by service provider was calculated for each district by multiplying the average expenditure per month per role by the numbers of service providers working in that district. Annual cost of human resources was then calculated by multiplying total monthly expenditure by 12, for each district.
- *Annual meetings*, *workshops*, *supervisions*: Expenditure on annual meetings, workshops, and supervisions were based on reported institutional expenditure and were divided by the number of UMSs in Iran (62), to be equally distributed to all medical universities.
- *Retraining and material revision*: The expenditures on retraining and material revision was detailed by design, content gathering, production, translation, and printing costs and their sum was divided by 5 as they were planned to be updated in 5-year cycles. These costs were also divided by 62.

#### C. Cost per Capita estimation

To make the cost of protocols comparable to one another, we adopted the strategy to standardize the costs with dividing the cost by the eligible population. Considering that we are not performing regression analysis, we did not transform the estimations to meet distribution requirements of regression. The eligible population for each protocol was extracted from the 2016 national census [23]. The costs were recategorized into variables and fixed costs; supplies and personnel were considered variable and other costs were considered fixed costs. It is important to note that some items included in the fixed costs category, particularly equipment should strictly be classified as ‘Mixed’ or “semi-variable” expenses. For the purposes of this paper, however, it was assumed that they would remain constant throughout the program’s implementation. Because the cost of program introduction are one-off costs and only applicable in the first year of implementation, the final costs are reported both incorporating and not incorporating them. Costs for delivery of IraPEN were estimated for individual protocols, carried out by first tagging each equipment and supply item with the service protocol it was used in. The expenditures on supplies and equipment used in multiple protocols, such as disposable gloves, were divided equally between those protocols. Laboratory costs incurred for analyzing cervical and colorectal cancer screening samples were also not included. The staffing costs for delivery of each service was estimated by calculating the cost of healthcare staffing per service using times per service as indicated in staffing time-sheets. Cost of physician visit was attributed to specific protocols accordingly. Non-specific costs, which are costs not related to a service (e.g., program introduction, supervision, and customization) were apportioned to each protocol equally. Fixed costs were divided by the total number of eligible individuals in each of the districts. Variable costs were divided by target population covered by the program. Total per capita cost per service was then calculated for each protocol by adding these figures.

All costs are reported in US$ using the official central bank of Iran exchange rate for 2015–2016 reported at US$1 = 36440 IRR [24]. The timeframe of analysis is annual.

In terms of technical limitations, institutional cost calculations relied fundamentally on the quality of data provided by entities responsible for planning and implementation of the program. Considerable efforts were made in accurate collection of expenditure data, such as requests for detailed cost breakdowns and financial document numbers. There may, however, be inaccuracies in the information provided, such as over-declaration of consumables used at the point of service. Additional efforts were made to address anomalous figures by reconfirmation with relevant sources. Determining the validity of supplied data beyond these efforts were outside the scope of this study. Several assumptions and simplifications, inevitable in research of this nature, were made in calculations. The number and length of visits per provider were based on timesheets collected specially for this study around the same dates. The primary simplification made here was to assume constant and continued demand for visits throughout the implementation period. The estimations, therefore, ignore variations in demand over time. The non-inclusion of laboratory costs for analyzing cervical and colorectal cancer screening samples was another limitation. This may significantly increase the costs associated with these two protocols.

### Summary

Taken together, and as depicted in Fig 1, the cost components were as follows:

• Annual Incremental cost per protocol, including
    ○ Fixed costs (costs that do not change with number of people), consisted of
        ■ Capital costs (on-off items)
            • *Initial meetings*, *workshops*, *consultancies*, *supervision*, *and customization*
                ○ *Done once and for the whole IraPEN*. *So*, *it was divided by the number of protocols to partition it between them*
        ■ Recurrent costs (consumables and to-be-regularly purchased items)
            • *Equipment*
                ○ $Annual\;cost\;of\;equipment=Number\;of\;purchased\;units\times \frac{Current\;unit\;cost}{annualization\;factor\;based\;on\;useful\;life\;time}$
                ○ $Annulization\;factor=\frac{{\left(1+discount\;rate\right)}^{useful\;life\;time}-1\;}{discount\;rate{\times (1+discount\;rate)}^{useful\;life\;time}}$
            • *Annual meetings*, *workshops*, *supervisions*
                ○ Reported Expenditure divided by the number of UMSs in Iran
            • *Retraining and material revision*
                ○ The expenditures detailed by design, content gathering, production, translation, and printing costs divided by 5 (because it was planned to repeat each 5 years) divided by the number of UMSs in Iran
    ○ Variable costs (costs that do not change with number of people), consisted of
        ■ Recurrent costs
            • *Supplies*
                ○ $Annual\;cost\;of\;supply=12\times \frac{Total\;cost\;of\;supply\;during\;pilot}{duration\;of\;pilot\;in\;month}$
            • *Personnel (community health workers*, *midwifes*, *and physicians)*
                ○ Annualpersonnelcost=percentageofdailyworktimespentfortheprogramperrole×averagemonthlysalaryofthepersonnelperrole×12
• Total eligible population to be covered by the protocol

Then we divided the incremental cost by the population covered and reported them as final results.

### Ethical considerations

This study was assessed by multiple organizations for ethical considerations. This study was reviewed and approved by Deputy of Health, Ministry of Health and Medical Education of Iran before the study began. Tehran University of Medical Sciences IRB approved of this study. Informed written consent was received and documented from the participants, after describing the details of the study. The need for consent was waived by the ethics committee to gather administrative data.

## Results

A total of 637 individuals, from 551 families, who met IraPEN eligibility criteria were interviewed, out of whom 265 individuals had received IraPEN services at least once, corresponding to an overall coverage of 40.74% in two districts. Naqadeh and Shahreza had a coverage of 46.1%, and 36.82%, respectively. Over 75% of participants living in rural areas were covered by IraPEN.

Total expenditures are presented in Table 1. Introduction cost only contributed to 2.07% of the total costs. The human resources and supplies had the highest contribution in the total program cost (74.97% and 15.76%, respectively). Expenditure on human resources also contributed the highest portion of cost of each protocol (S2 Table). The cost categories with lowest contribution were retraining followed by customization.

**Table 1:** Summary of annual costs of IraPEN pilot program in Naqadeh and Shahreza (US$).

	Variable costs				Fixed costs							
Protocol	Recurrent cost per protocol per year				Capital cost per protocol per year		Recurrent cost per protocol per year			Capital cost per protocol per year		
	Supply	Share of supply cost in each protocol (%)	Personnel	Share of personnel cost in each protocol (%)	Equipment	Share of equipment cost in each protocol (%)	Consultancy	Retraining	Supervision	Introduction	Customization	Supervision
MI & Stroke prevention	102859.24	72.26	186455.94	27.55	7389.52	82.96	4291.00	395.87	3348.26	3754.37	1482.81	1654.70
Respiratory diseases screening	671.95	0.47	37361.09	5.52	1513.00	16.99	4291.00	395.87	3348.26	3754.37	1482.81	1654.70
CRC prevention	7413.57	5.21	81284.81	12.01	0.00	0.00	4291.00	395.87	3348.26	3754.37	1482.81	1654.70
Female cancer prevention	31270.27	21.97	163802.66	24.20	5.04	0.06	4291.00	395.87	3348.26	3754.37	1482.81	1654.70
NCD risk factors survey	129.39	0.09	207893.64	30.72	0.00	0.00	4291.00	395.87	3348.26	3754.37	1482.81	1654.70
Total	142344.42		676798.14		8907.55		21455.02	1979.39	16741.34	18771.88	7414.07	8273.52
Share of each cost category from total cost of IraPEN implementation (%)	15.77		74.98		0.99		2.38	0.22	1.85	2.08	0.82	0.92

When focusing on the proportion of each program in equipment, personnel, and supplies MI & Stroke prevention had the highest share of supplies and equipment costs and NCD risk factors survey had the highest share of personnel costs. Interestingly, NCD risk factors survey was the protocol with the lowest share in supplies and equipment costs (Table 1).

The final incremental costs of the IraPEN implementation per user, with and without introduction cost, were US$24.90 and US$25.32, respectively. The total incremental cost per capita for each protocol ranged between US$1.05 to US$7.45. Among all protocols, MI & Stroke prevention protocol had the highest cost per user followed by NCD risk factors survey (Table 2). The protocol with the lowest cost was respiratory diseases screening.

**Table 2:** Costs per protocol per capita categorized by fixed and variable costs (US$).

	Variable costs		Fixed costs							Total	
Protocol	Recurrent		Capital	Recurrent			Capital				
	Supply	Personnel	Equipment	Consultancy	Retraining	Supervision	Introduction	Customization	Supervision	Without introduction cost	with introduction cost
MI & Stroke prevention	2.33	9.47	0.16	0.10	0.008	0.07	0.09	0.03	0.04	7.45	7.53
Respiratory diseases screening	0.03	1.86	0.03	0.10	0.008	0.07	0.09	0.03	0.04	1.05	1.13
CRC prevention	0.63	6.89	0.00	0.10	0.008	0.07	0.09	0.03	0.04	3.95	4.03
Female cancer prevention	2.20	11.58	0.00	0.10	0.008	0.07	0.09	0.03	0.04	7.13	7.23
NCD risk factors survey	0.01	10.42	0.00	0.10	0.008	0.07	0.09	0.03	0.04	5.32	5.40
Total	5.21	40.22	0.19	0.52	0.04	0.37	0.49	0.19	0.23	24.91	25.33

## Discussion

This research aimed to estimate the incremental cost of PEN implementation in Iran from healthcare provider perspective. The results show human resources to have had the highest contribution, followed by supplies. The final incremental costs of the IraPEN implementation per capita, excluding the cost of program introduction, was US$24.90. Total incremental cost per capita for each protocol ranged between US$1.05 and US$7.45, with MI & Stroke prevention protocol being the highest and respiratory diseases screening protocol being the lowest. The highest cost of supplies and equipment were for MI & Stroke prevention protocol and the highest cost of personnel was for NCDs risk factors survey protocol.

Our study is one of the first to evaluate the incremental implementation cost of health interventions proposed by PEN program in primary health care settings in a middle-income country [16]. Original WHO cost estimates for implementing the PEN’s package of “best-buy” s, was less than US$3 per capita in a UMIC, much lower than our US$24.90 figure for IraPEN [2, 25–27]. Best-buys should constitute a small proportion of the overall health spending of a country, less than 1% in upper middle-income countries [27]. Our results show the cost of IraPEN package to be 6.80% of the annual per capita healthcare expenditure in Iran [28], and can thus can be considered high-cost. The observed differences could be generally due to two reasons of accuracy and context. First, WHO cost calculations are mainly based on the total expected cost of implementation and in many cases, there is lack of accurate data and the gap is filled by expert estimations [29]. Additionally, WHO calculations are based on predicted costs, whereas we assessed the realized costs of implementation. Therefore, we consider the approach of this study to be accurate and thus more valid. The differences could come from the particular context of the study. Nevertheless, we expect not this to be the case because WHO considers Iran an upper-middle income country and incorporates the characteristics of upper-middle income countries in recommendations. And finally, and as depicted later in the discussion, WHO do not recommend detailed implementation. Therefore, a part of the difference could come from lack of efficiency of the IraPEN implementation and this is why we believe with configurations in the proposed plan, we could get closer to WHO target.

Nevertheless, this costing report can be considered as best estimate currently available for implementing a PEN variant in an MIC. Our study was the first attempt at gathering and presenting empirical evidence regarding the cost of implementing a PEN-based program. We took ingredients approach of costing for supplies, human resources, and equipment which is considered to be more precise compared to other methods like reference costing [22, 30, 31]. The results may offer empirical inputs for macroeconomic costing tools, such as OneHealth, for more realistic cost projections of PEN program in the future and in different countries. The information presented here may also be used internationally by policymakers to estimate the budgets necessary for implementing PEN in settings with previously established primary health care system.

Our study faced some limitations which should be discussed in brief. On top of all, though cost matters, an important factor to consider in making the decision about implementation of an intervention or a policy is the perspective of the patients and healthcare deputies. Due to the financial limitations and being out of the scope of the main goal in the study design, we did not systematically record the perspectives of the stakeholders. Nevertheless, we could infer that a great part of the opinion about the program for healthcare deputies is formed by the cost of the program in our informal discussion policy makers and decision makers in MoHME, UMSs, and DHSs. Additionally, the process of IraPEN implementation did not change the type and configuration of the service provision to patients in the sense that they did notice any big change in the healthcare center and service provision very likely stayed the same in their eyes. This design helped us in two matters. First, we are confident that the measurements are not affected by the patients’ expected outcomes of the changes. Additionally, as patients probably did not notice any difference, their perspective is very likely to be the same before and after the implementation of IraPEN. Therefore, the decision about continuing or halting the program could put patients’ perspective aside in further decision-making steps.

Our estimations just covered incremental cost of implementation in the Iranian healthcare context. The total cost of the program is required to be calculated for further studies and considerations. Nevertheless, and considering all strengths and limitations, our findings could be utilized in low and upper-middle income countries with limited resources and funding to inform the requirements for development and implementation of low cost and effective interventions for prevention and control of NCDs. Based on our findings we suggest some strategies to reduce the cost of the PEN program implementation in the Iranian context as well as other settings: 1) Because the human resource training, preparation, wages, and their supervision contributed the most of the program implementation cost, we suggest alterations of the current program to reduce the necessity and role of humans. Inserting the low-cost innovative technology to the chain of care have shown to reduction and efficiency improvements [32–35]. A primary example of this would be to utilize eHealth strategies in CVD risk calculation and consequent education on associated behavioral risk factors. In addition, any future performance monitoring system and cost reduction strategy requires the relevant data to be collected. Therefore, further strengthening of electronic record keeping for the program will assist in gathering valuable intelligence regarding local needs besides cost reduction. Moreover, it allows for customizable resource planning and allocation of the program which would help furtherly with increasing the efficiency of the program and decreasing its cost. 2) Restriction of the program’s inclusion criteria to target individuals at higher risk of NCDs will lower cost of human resources and the required materials as well. Provision of services could be planned according to the disease’s prevalence and their relative burden in the population. For instance, the incidence rate of cervical and colorectal cancers in the Iranian population was, respectively, 3.2 and 7.65 in 100,000 in 2015 [36]. It therefore seems that the colorectal cancer poses a greater population risk than cervical cancer and should be thus prioritized. Additionally, the cost-effectiveness of these early screening interventions will need to be thoroughly considered and funds should be prioritized and allocated accordingly. 3) Besides direct interventions for patients from healthcare providers, there is a need for exploration of more innovative behavioral approaches, targeted at the reduction of NCDs risk factors at the public and individual levels [27, 37]. It should however be noted that such interventions, while relatively straightforward to initiate, are shown to have mixed records of success [38, 39] and require careful review of the available evidence prior to making and implementing policy decisions [40].

## Conclusions

Taken together, the present study showed that the IraPEN program implementation is considered a high-cost package in Iran. It is, however, difficult to make decisions on the implementation of NCD prevention and control programs purely based on their cost. Informed decision making, in this regard, requires an assessment of a programs’ effectiveness at national and subnational levels. Moreover, justifications and alterations to the current package could reduce the costs, leading to increased efficiency of the program, in the similar contexts comparing to Iran.

## Supporting information

S1 Table Description of cost items for IraPEN implementation in the pilot phase. (DOCX)

S2 Table Estimated total and per protocol expenditure on staffing by service provider (US$). (DOCX)
