# Supplementary material for: The incremental cost of implementing the world health organization Package of essential non-communicable (PEN) diseases interventions in Iran
Source: PLOS Glob Public Health. 2023 Feb 8;3(2):e0000449. doi: 10.1371/journal.pgph.0000449 (PMC10021820; doi:10.1371/journal.pgph.0000449)
Supplement: S1 Table — (DOCX) [file pgph.0000449.s001.docx]

| *S1 Table. Description of cost items for IraPEN implementation in the pilot phase* | | | | | | | | |
| --- | --- | --- | --- | --- | --- | --- | --- | --- |
| Capital costs | | | | **Recurrent costs** | | | | |
| Fixed | | | | | | | **Variable** | |
| Non-specific costs | | | **Specific costs** | **Non-specific costs** | | | **Specific costs** | |
| Introduction | Customisation | National Supervision | Equipment | Consultation | Retraining and material revision | Local Supervision | Supplies | Human resource |
| - Overseas meetings - Domestic meetings - Overseas workshops - Domestic workshops - International consultancy - Domestic consultancy | - Protocol - Guidelines - Forms & questionnaires - Training materials - Software - Website | Supervision during the establishment phase by international, national, and provincial agencies | Equipment purchased specifically to deliver IraPEN services | - Domestic meetings - Domestic workshops - Domestic consultancy | - Protocol - Guidelines - Forms & questionnaires - Training materials - Software - Website | Supervision during the establishment phase by international, national, and provincial agencies. | Consumable items purchased specifically for delivering IraPEN services | salary payments attributable to delivering IraPEN per role |
