# Supplementary material for: The incremental cost of implementing the world health organization Package of essential non-communicable (PEN) diseases interventions in Iran
Source: PLOS Glob Public Health. 2023 Feb 8;3(2):e0000449. doi: 10.1371/journal.pgph.0000449 (PMC10021820; doi:10.1371/journal.pgph.0000449)
Supplement: S2 Table — (DOCX) [file pgph.0000449.s002.docx]

| *S2 Table. Estimated total and per protocol expenditure on staffing by service provider (US$)* | | | |
| --- | --- | --- | --- |
| Protocol | **CHW** | **Physician** | **Midwife** |
| MI & Stroke prevention | 144242.60 | 42213.34 | 0.00 |
| Respiratory diseases screening | 28255.65 | 9105.44 | 0.00 |
| CRC prevention | 61612.70 | 19672.12 | 0.00 |
| Female cancer prevention | 27833.66 | 9330.97 | 126638.03 |
| NCD risk factors survey | 158469.50 | 49424.15 | 0.00 |
| Total | **420414.10** | **129746.02** | **126638.03** |
